# Supplementary material for: Noninvasive and reliable automated detection of spreading depolarization in severe traumatic brain injury using scalp EEG
Source: Commun Med (Lond). 2023 Aug 19;3:113. doi: 10.1038/s43856-023-00344-3 (PMC10439895; doi:10.1038/s43856-023-00344-3)
Supplement: Supplementary file 1 — Supplementary Information [file 43856_2023_344_MOESM1_ESM.pdf]

Supplementary Materials:
Noninvasive and reliable automated detection of spreading depolarization in severe traumatic brain injury using scalp EEG

Alireza Chamanzar[1,2*], Jonathan Elmer[3], Lori Shutter[4], Jed Hartings[5], and Pulkit Grover[1,2*]
[1]Electrical and Computer Engineering Department, Carnegie Mellon University, Pittsburgh, PA, USA.
[2]Neuroscience Institute, Carnegie Mellon University, Pittsburgh, PA, USA.
[3]Departments of Emergency Medicine, Critical Care Medicine and Neurology,
University of Pittsburgh School of Medicine, Pittsburgh, PA, USA.
[4]Department of Critical Care Medicine, Neurology and Neurosurgery,
University of Pittsburgh School of Medicine, Pittsburgh, PA, USA.
[5]Department of Neurosurgery, University of Cincinnati, Cincinnati, OH, USA.
[*]correspondence to: {pgrover, achamanz}@andrew.cmu.edu.

This file includes:

- **Supplementary Note 1-2**
- **Supplementary Fig. 1-7**
- **Supplementary Table I**
- **Supplementary References**

Supplementary Note 1. Hemispheric comparison of scalp EEG power in patients with craniectomy

As shown in Fig. 2 in our paper, visual inspection of continuous EEG signals in patients with craniectomy indicates baseline power enhancement in the ipsilateral hemisphere (the hemisphere with a missing piece of skull). To quantify this power enhancement, we performed a hemispheric comparison of average baseline power for the 12 patients: (i) we chose non-overlapping 4-hour windows of scalp EEG recordings. The length of windows were arbitrarily chosen, but long enough for average power estimation and short enough not to get affected by the baseline power variation over time, (ii) for each window, the average baseline power was estimated at each scalp electrode, (iii) the estimated baseline powers were averaged across ipsilateral and contralateral electrodes, (iv) for each window, the difference between ipsilateral and contralateral average powers were normalized by the contralateral power ($\Delta P = \frac{\overline{P}_{ipsi} - \overline{P}_{contra}}{\overline{P}_{contra}}$). This normalization helps to exclude the effect of baseline power variations across time windows and provides a measure of hemispheric power difference, i.e., ΔP, and (v) finally, we performed a non-parametric statistical test using Wilcoxon T-test[1], to assess the statistical significance of deviation of P from zero. Based on the results, for all of the 12 patients in the dataset, there is a significant difference ($p < 1e-8$) between the ipsilateral and contralateral average power ($\overline{P}_{contra} < \overline{P}_{ipsi}$). The results are consistent with our visual inspection of the baseline powers in ipsilateral and contralateral electrodes and the concept of "breach rhythm" reported in the literature, which is an increase in signal power in a wide range of frequencies in areas with skull defects[2,3]. The average power across all patients is 488.2 ± 68.1 (μV^2) and 341.7 ± 39.8 (μV^2) for ipsilateral and contralateral hemispheres respectively. Supplementary Fig.1 shows the histograms of $\overline{P}_{contra}$ (blue) and $\overline{P}_{ipsi}$ (green) across the 196 windows for all of the 12 patients in the dataset.

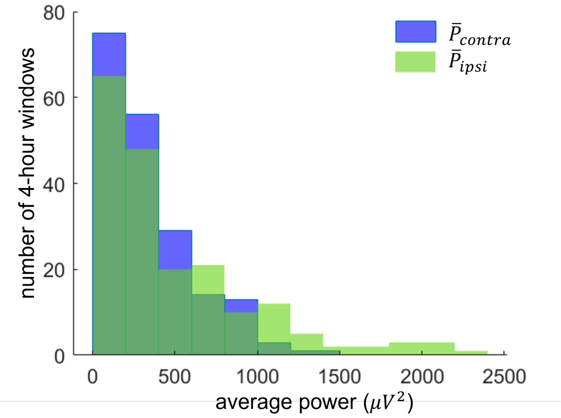

Supplementary Fig. 1. **Hemispheric comparison of scalp electroencephalography (EEG) power in patients with craniectomy.** Histograms of scalp average power in the contralateral hemisphere (i.e., $\overline{P}_{contra}$, the hemipshere with intact skull, shown in blue) and ipsilateral hemisphere (i.e., $\overline{P}_{ipsi}$, the hemisphere with craniectomy, shown in green) across the 196 windows for all of the 12 patients in the dataset.

Supplementary Note 2. Discussions on real-time monitoring of SDs

Real-time monitoring of patients with brain injuries is crucial to predict and prevent worsening brain injuries through SD detections at ICUs. Our modified WAVEFRONT algorithm in this paper can provide SD detection results for each 4-hour time window (epoch) with only 5min computational delay.

In this paper, we made significant improvements in the speed of SD detection using WAVEFRONT: (i) in the preprocessing pipeline, the ICA analysis was accelerated significantly, by 25 folds, using a GPU implementation of this function (i.e., CUDAICA[4]). Therefore, preprocessing and pruning a 10-hour scalp EEG recording in this dataset takes minutes (∼5min) using a GPU, in comparison to the CPU processing speed (∼2 hours); (ii) the main steps of WAVEFRONT were accelerated using parallel processing and distribution across multiple CPU physical cores (64 CPU cores using AMD Ryzen Threadripper PRO 3995WX). E.g., the outlier rejection step was done in parallel for all of the 19 EEG scalp channels and 6 ECoG channels. In addition, SD detection in the main step of the algorithm (including Cylindrical projection, Interpolation and thresholding, Subsampling and optical flow calculation, Quantization of orientations, Orientations bounding boxes (OBBox), and Stitching process and the final decision on detection) was done in parallel for all of the epochs of each patient, up to 64 epochs at the same time (upper bounded by the number of available CPU cores). Once the algorithm is trained and the optimal set of parameters are found, it only takes a few seconds (<5s) to perform SD detection in each epoch of 240min of data. This enables the algorithm to provide SD detection results for each recording epoch, with negligible computational delay (∼5min, including the preprocessing

time): every 3 hours (epoch step size in our method), WAVEFRONT provides SD detection results. This long detection interval is required to capture the spatiotemporal dynamics of slowly propagating SD waves (1-8 mm/min). The processing performance numbers are provided using a workstation with 64 CPU cores using AMD Ryzen Threadripper PRO 3995WX, 512GB RAM, and using MATLAB R2018b in Windows 10 Enterprise.

Supplementary Table I. Search grids for the WAVEFRONT's parameters.

Parameter	Search grid
Thr_1	$[0.1, 0.14, 0.18, \cdots, 0.9]$
Thr_2	$[0.05, 0.10, 0.15, \cdots, 0.8]$
Thr_3	$[2, 3, 4, \cdots, 10]$
Thr_4	[0.6,0.7,0.8,0.9]

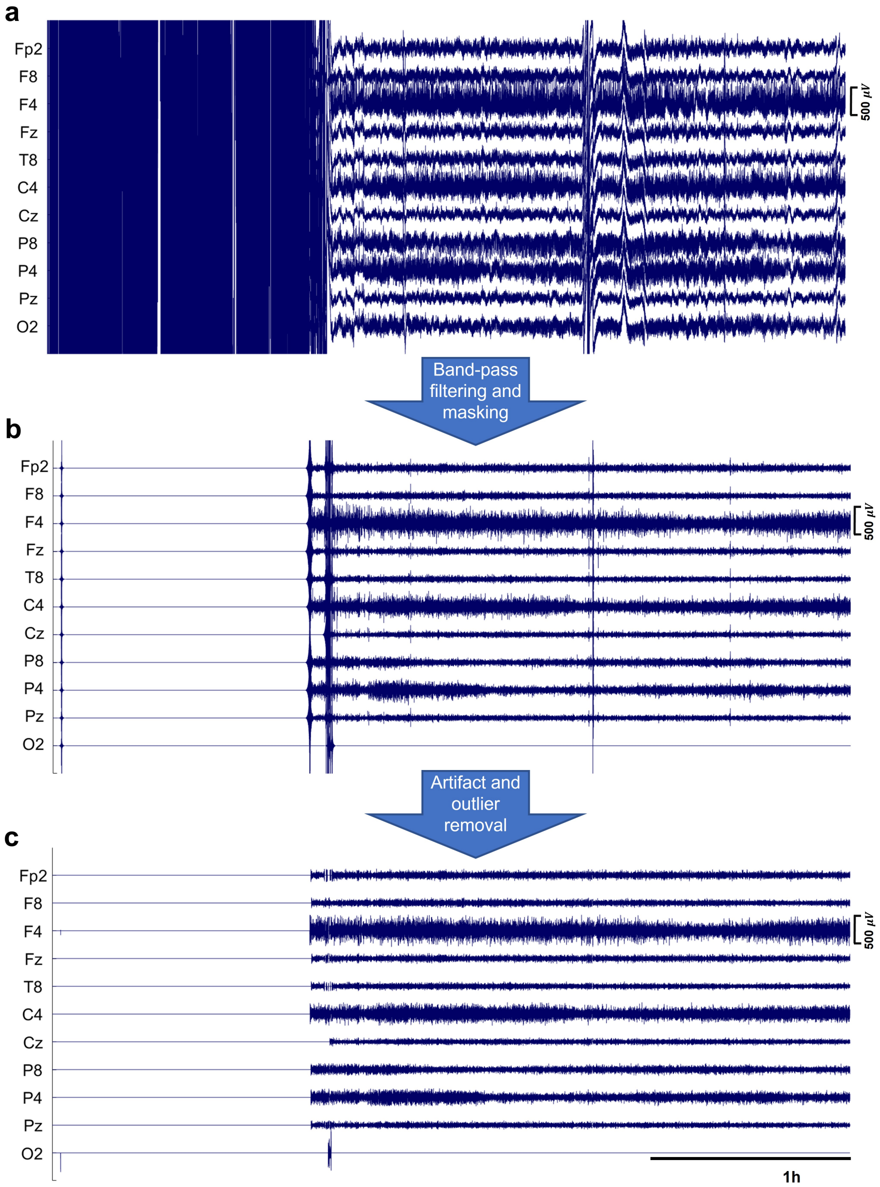

Supplementary Fig. 2. **EEG preprocessing steps.** Application of the preprocessing steps on the ipsilateral electroencephalography (EEG) recordings of a patient with right decompressive hemicraniectomy (DHC), in a 4-hour time window: a) the full-band EEG signals, with poor quality (high impedance) in the first $\sim$ 77min of the recording, which is masked out in the band-pass filtered signals (Delta band, [0.5,4] Hz) shown in (b). The artifacts and outliers are then detected and removed in (c).

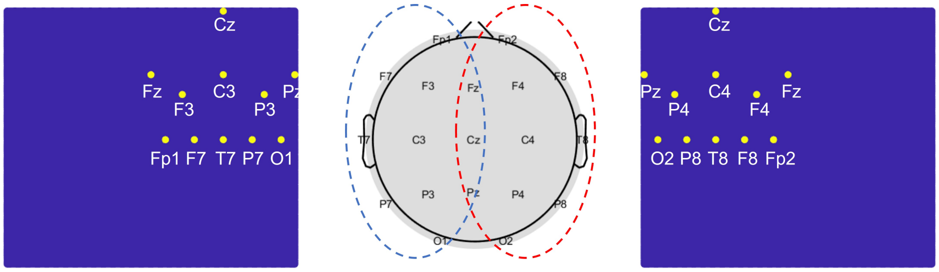

Supplementary Fig. 3. **Ipsilateral scalp electrode locations in 2D images.** Ipsilateral scalp electrode locations in the 2D images, resulting from the cylindrical projection step in WAVEFRONT (e.g., I_{Sparse}, I_{Smooth}, and I_{BW}), for patients with left (left panel) and right (right panel) decompressive hemicraniectomy (DHC).

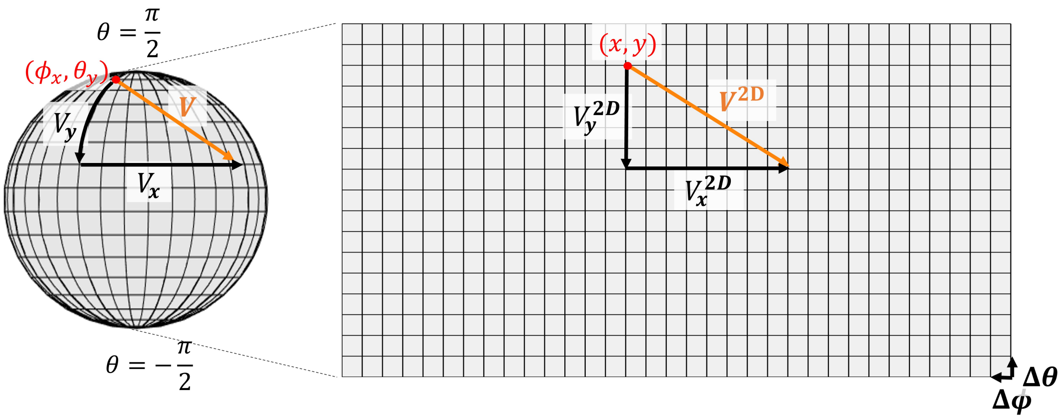

Supplementary Fig. 4. **Mapping of optical flow on the scalp spherical surface.** Mapping of an optical flow ($\mathbf{V}^{2D}(x,y)$) in the 2D plane on the scalp spherical surface ($\mathbf{V}(\phi_x, \theta_y)$).

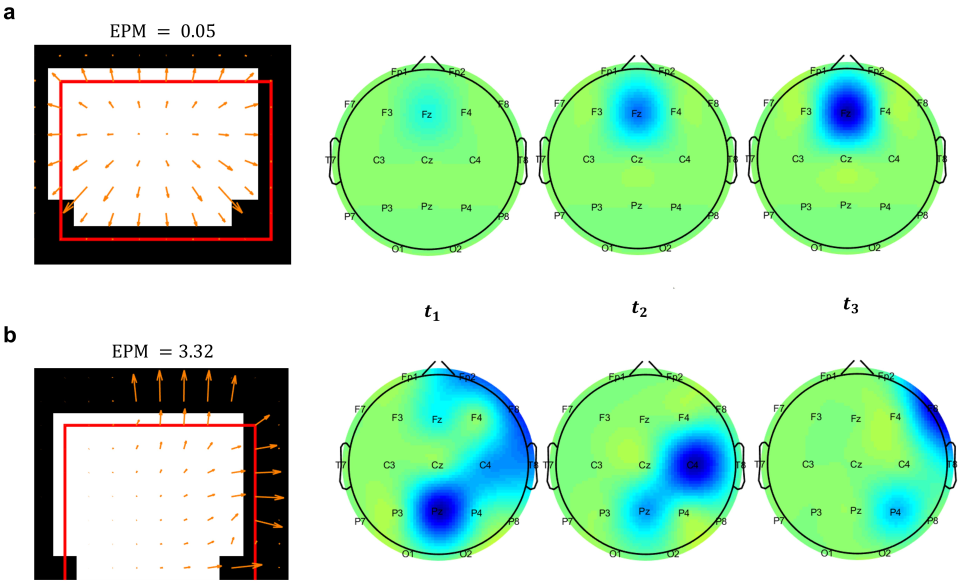

Supplementary Fig. 5. **Effective propagation measures (EPMs).** a) a bounding box (BBox, red box) with a pop-up transition (EPM= 0.05), and b) a BBox with significant effective propagation (EPM= 3.32), along with their corresponding depression transitions on scalp (dark blue spots in the scalp topography) at three time points.

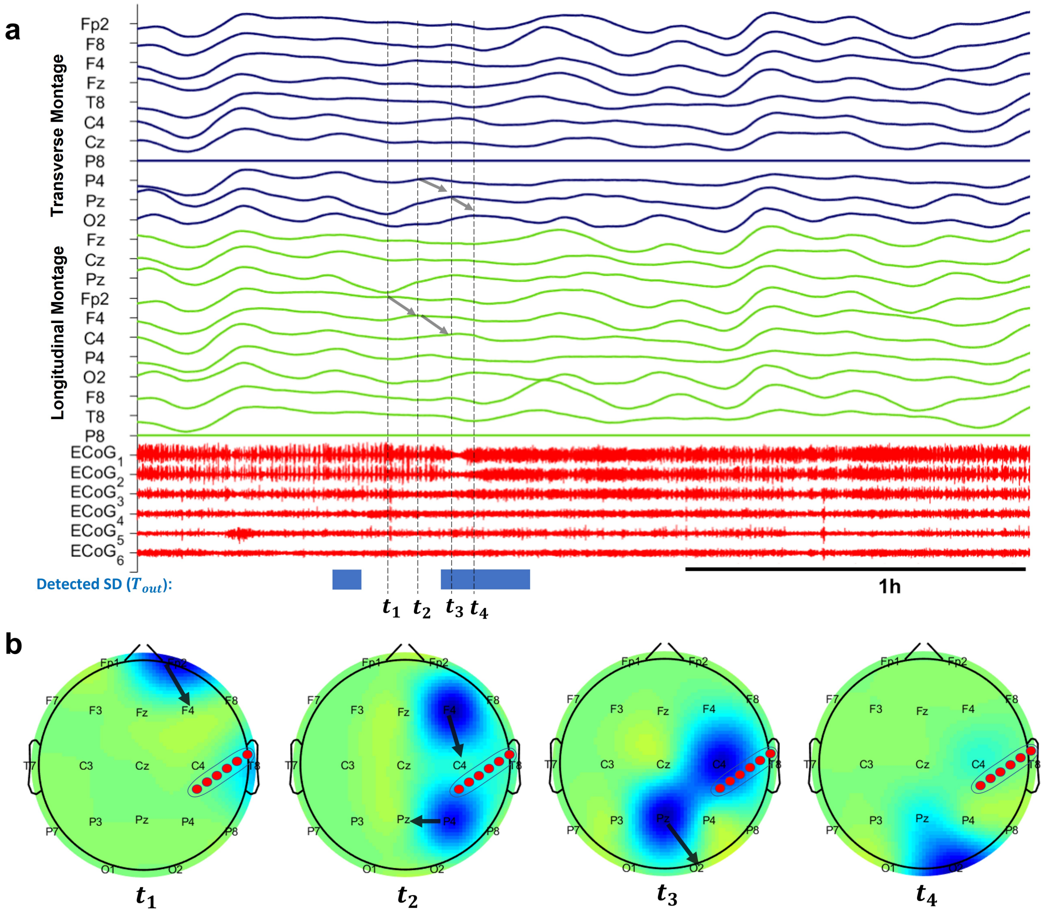

Supplementary Fig. 6. **Visualization of a single isolated SD event in a patient right DHC.** Visualization of a single isolated spreading depolarization (SD) event in patient 6 with right decompressive hemicraniectomy (DHC) (see the computed tomography (CT) scan of this patient in Fig. 1 in our paper): a) time traces of S_{Xcorr} and electrocorticography (ECoG) signals, where four time-points of the selected SD event are marked as t_1, t_2, t_3, and t_4 with maximum depressions at Fp2, (F4,P4), (C4,Pz), and O2 respectively, b) scalp topography of SD depressions at the four corresponding time points. The intracranial ECoG strip is located around the right temporoparietal lobe. The detected events ($T_{out} = 1$) using WAVEFRONT are marked with blue strips in (a).

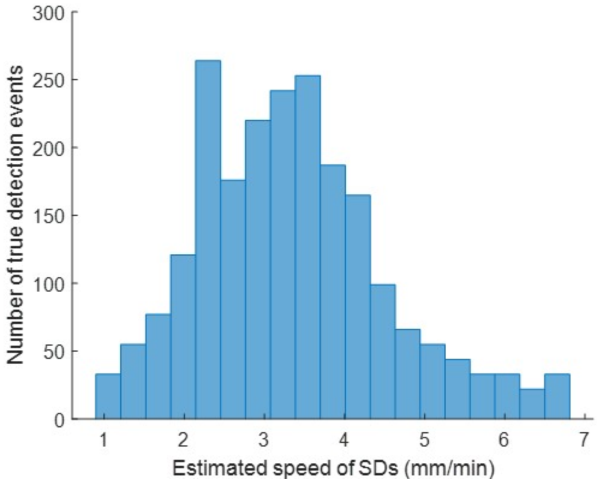

Supplementary Fig. 7. **Histogram of the estimated speed of propagation of detected spreading depolarization (SD) events in the scalp electroencephalography (EEG) Delta band.** The speed of propagation of SD waves ranges from 0.9 to 6.8 mm/min, with the maximum population around 3.6 mm/min for 12 severe traumatic brain injury (TBI) patients in the dataset.

Supplementary References

[1] Frank Wilcoxon. Individual comparisons by ranking methods. Springer, 1992.

[2] WA Cobb, RJ Guiloff, and Janice Cast. Breach rhythm: the eeg related to skull defects. Electroencephalography and clinical neurophysiology, 47(3):251–271, 1979.

[3] Francesco Brigo, Rosario Cicero, Antonio Fiaschi, and Luigi Giuseppe Bongiovanni. The breach rhythm. Clinical neurophysiology, 122(11):2116–2120, 2011.

[4] Federico Raimondo, Juan E Kamienkowski, Mariano Sigman, and Diego Fernandez Slezak. CUDAICA: GPU optimization of infomax-ICA EEG analysis. Computational intelligence and neuroscience, 2012:2–2, 2012.
