# Supplementary material for: Noninvasive and reliable automated detection of spreading depolarization in severe traumatic brain injury using scalp EEG
Source: Commun Med (Lond). 2023 Aug 19;3:113. doi: 10.1038/s43856-023-00344-3 (PMC10439895; doi:10.1038/s43856-023-00344-3)
Supplement: Supplementary file 4 — Description of Additional Supplementary Files [file 43856_2023_344_MOESM4_ESM.pdf]

Description of Additional Supplementary Files

File Name: Supplementary Data 1

Description: Supplementary Data 1 contains source data for the main figures with numerical results in this paper.
