# Supplementary material for: Noninvasive and reliable automated detection of spreading depolarization in severe traumatic brain injury using scalp EEG
Source: Commun Med (Lond). 2023 Aug 19;3:113. doi: 10.1038/s43856-023-00344-3 (PMC10439895; doi:10.1038/s43856-023-00344-3)
Supplement: Supplementary file 5 — Reporting Summary [file 43856_2023_344_MOESM5_ESM.pdf]

nature portfolio

Corresponding author(s): Alireza Chamanzar, PhD

Last updated by author(s): Aug 3, 2023

Reporting Summary

Nature Portfolio wishes to improve the reproducibility of the work that we publish. This form provides structure for consistency and transparency in reporting. For further information on Nature Portfolio policies, see our Editorial Policies and the Editorial Policy Checklist.

Statistics

For all statistical analyses, confirm that the following items are present in the figure legend, table legend, main text, or Methods section.

n/a	Confirmed	
☐	☒	The exact sample size (n) for each experimental group/condition, given as a discrete number and unit of measurement
☐	☒	A statement on whether measurements were taken from distinct samples or whether the same sample was measured repeatedly
☐	☒	The statistical test(s) used AND whether they are one- or two-sided<br>*Only common tests should be described solely by name; describe more complex techniques in the Methods section.*
☐	☒	A description of all covariates tested
☐	☒	A description of any assumptions or corrections, such as tests of normality and adjustment for multiple comparisons
☐	☒	A full description of the statistical parameters including central tendency (e.g. means) or other basic estimates (e.g. regression coefficient) AND variation (e.g. standard deviation) or associated estimates of uncertainty (e.g. confidence intervals)
☒	☐	For null hypothesis testing, the test statistic (e.g. F, t, r) with confidence intervals, effect sizes, degrees of freedom and P value noted<br>*Give P values as exact values whenever suitable.*
☒	☐	For Bayesian analysis, information on the choice of priors and Markov chain Monte Carlo settings
☒	☐	For hierarchical and complex designs, identification of the appropriate level for tests and full reporting of outcomes
☐	☒	Estimates of effect sizes (e.g. Cohen's d, Pearson's r), indicating how they were calculated

Our web collection on statistics for biologists contains articles on many of the points above.

Software and code

Policy information about availability of computer code

Data collection	The dataset we used was obtained as part of a multicenter clinical study that monitored SDs in TBI patients (ClinicalTrials.gov Protocol ID: 08-96-12-01). Continuous EEG signals were recorded over a few days (95±42.2 hours on average) following DHC using a DC-coupled EEG amplifier (CNS Advanced ICU EEG Amplifier from MOBERG ICU Solutions), with a sampling frequency of 256Hz, from 19 electrodes placed at 10-20 standard locations. All of the EEG electrodes were referenced to a common reference electrode. CNS Advanced ICU EEG software was used to collect and convert the dataset.
Data analysis	We preprocessed the recorded EEG signals using EEGLAB toolbox (v2019.0) in MATLAB. WAVEFRONT was developed in MATLAB (R2018b), using standard toolboxes, and EEGLAB toolbox (v2019.0)43 . All MATLAB code is made available online on GitHub (DOI: 10.5281/ zenodo.8210380).

For manuscripts utilizing custom algorithms or software that are central to the research but not yet described in published literature, software must be made available to editors and reviewers. We strongly encourage code deposition in a community repository (e.g. GitHub). See the Nature Portfolio guidelines for submitting code & software for further information.

Data

Policy information about availability of data

All manuscripts must include a data availability statement. This statement should provide the following information, where applicable:

- Accession codes, unique identifiers, or web links for publicly available datasets
- A description of any restrictions on data availability
- For clinical datasets or third party data, please ensure that the statement adheres to our policy

Supplementary Data 1 contains source data for the main figures with numerical results in this paper. The dataset was obtained as part of a multicenter clinical study, ClinicalTrials.gov ID number NCT00803036. The anonymized raw EEG dataset may be made available upon request, contingent upon contractual obligations and data sharing and cooperative agreements.

Human research participants

Policy information about studies involving human research participants and Sex and Gender in Research.

Reporting on sex and gender	[full info is available in the study protocol provided before]<br><br>All patients who present to the study hospitals for treatment of TBI were screened for study eligibility. The subject population includes all races, ethnicities, and sex/gender groups. There was no selection criteria based on race, ethnicity, sex, or gender. The subject population reflects the demographics of the catchment areas of the participating study sites. The proposed study was conducted in the adult intensive care unit to include those patients aged ≥18 years seeking treatment for TBI. Inclusion and exclusion criteria are described in section E.2 in the study protocol.<br>No special populations were to be included in this research, such as children, prisoners or pregnant women. A separate, age-specific study in children would be required due to logistic considerations of conducting this study in adult ICUs. The data obtained from this study may provide justification to warrant extension of similar, future studies to additional populations.
Population characteristics	[full info is available in the study protocol provided before]<br>There were no selection criteria based on race, ethnicity, sex, or gender. The subject population reflects the demographics of the catchment areas of the participating study sites. The proposed study were conducted in the adult intensive care unit to include those patients aged ≥18 years seeking treatment for TBI. Inclusion and exclusion criteria were described in section E.2 in the study protocol.<br>No special populations were to be included in this research, such as children, prisoners or pregnant women. A separate, age-specific study in children would be required due to logistic considerations of conducting this study in adult ICUs. The data obtained from this study may provide justification to warrant extension of similar, future studies to additional populations.
Recruitment	[full info is available in the study protocol provided before]<br>Recruitment materials and promotional advertisements were not appropriate and were not used for the study. Potential subjects admitted to each of the study sites were identified by study personnel through review of medical records, trauma logs and triage notes as well as on-duty doctors and nurses. When a potential subject was identified and had been screened against primary inclusion/exclusion criteria, they were approached about study participation.
Ethics oversight	All procedures were approved by the University of Cincinnati Institutional Review Board. A legally authorized representative for each patient provided surrogate consent for participation in the initial research study.

Note that full information on the approval of the study protocol must also be provided in the manuscript.

Field-specific reporting

Please select the one below that is the best fit for your research. If you are not sure, read the appropriate sections before making your selection.

☒ Life sciences ☐ Behavioural & social sciences ☐ Ecological, evolutionary & environmental sciences

For a reference copy of the document with all sections, see nature.com/documents/nr-reporting-summary-flat.pdf

Life sciences study design

All studies must disclose on these points even when the disclosure is negative.

Sample size	Data from 12 (9 male and 3 female) severe TBI patients were utilized in our study. Two patients had DHC in the left hemisphere and the remaining 10 patients had DHC in the right hemisphere. Eleven patients experienced subdural hematoma (SDH), and one patient had an epidural hematoma (EDH). Detailed information about these patients is included in Table I in the manuscript. Reliable validation performance is an indication of sufficient sample size in the train set. Due to the small number of patients in this study, overfitting to the available SD events is inevitable. We expect WAVEFRONT to achieve a better average validation performance by using a larger dataset of TBI patients with multiple SD events across different varieties of propagation patterns (single-gyrus, semi-planar, ring-shape, etc.), different ranges of

propagation speeds, and in different brain regions. In addition, we would be able to provide statistical guarantees for the detection and discrimination results using a larger dataset

Data exclusions	[see Results in the manuscript for more info] We also define Qavg, a measure of signal quality for each (sliding, 2 min) time window, as the number of electrodes, averaged over the 2-min interval, that are not masked out in the window over the hemisphere with DHC. Since there are 11 electrodes ipsilateral to the site of ECoG placement (see Fig. 2a, we include electrodes on the midline in the ipsilateral set), we choose a threshold of Qavg ≥ 6 for defining whether a time-window has good-quality recordings. Thus, time windows with Qavg < 6 are excluded from the SD detection performance calculations. In all, there were 36,709 excluded poor-quality windows (approximately 28% of the windows) across 12 patients. This large number of poor-quality windows is mainly due to the long time intervals during which the patients are disconnected from the EEG amplifier for procedures or imaging. During these intervals, the recordings were not stopped. Other poor-quality intervals may be, in part, due to the inherent limitations of scalp EEG recordings, e.g., low-density of EEG electrodes (only 11 ipsilateral) at ICUs increases the chance of recording intervals with almost no reliable EEG signal.
Replication	Leave-2-out cross-validation: To evaluate the generalizability of WAVEFRONT, and detect and prevent overfitting of our algorithm, we use cross-validation: we split the dataset into sets of train and validation patient groups, find the optimal sets of parameters for WAVEFRONT on the train sets, assess the SD detection performance on the validation sets, and average the performance on different validation sets. We choose two patients out of the total 12 patients, and leave them out for validation, in 66 different ways. Based on the results, using Delta frequency band scalp EEG, WAVEFRONT achieves a reliable average validation performance of TPR= 0.74 ± 0.03, with FPR< 0.015 (0.0145 ± 7.57 × 10−4). All of the reported results are in 95% confidence intervals.
Randomization	Exhaustive cross-validation was performed and the performance was averaged across all possible choices of validation set (66 different sets).
Blinding	The investigator was not blinded to group allocation during the data collection/analysis. Blinding was not relevant because the main purpose of the study was to show the feasibility of automated SD detection using scalp electroencephalography (EEG) and to test the performance of the WAVEFRONT algorithm.

Reporting for specific materials, systems and methods

We require information from authors about some types of materials, experimental systems and methods used in many studies. Here, indicate whether each material, system or method listed is relevant to your study. If you are not sure if a list item applies to your research, read the appropriate section before selecting a response.

Materials & experimental systems

n/a	Involved in the study
☒	☐ Antibodies
☒	☐ Eukaryotic cell lines
☒	☐ Palaeontology and archaeology
☒	☐ Animals and other organisms
☐	☒ Clinical data
☒	☐ Dual use research of concern

Methods

n/a	Involved in the study
☒	☐ ChIP-seq
☒	☐ Flow cytometry
☒	☐ MRI-based neuroimaging

Clinical data

Policy information about clinical studies

All manuscripts should comply with the ICMJE guidelines for publication of clinical research and a completed CONSORT checklist must be included with all submissions.

Clinical trial registration	ClinicalTrials.gov Protocol ID: 08-96-12-01
Study protocol	Protocol ID: 08-96-12-01
Data collection	The collection and recording of screening, physiological, surgical, radiological and chart extraction data that was performed for research purposes; placement of ECoG/EEG electrodes and ECoG/EEG monitoring at sites where these procedures were performed for research purposes. The patient and/or their health insurance was charged in the standard manner for services and procedures provided for their routine care.<br><br>Recruitment materials and promotional advertisements were not appropriate and were not used for the study. Potential subjects admitted to each of the study sites were identified by study personnel through review of medical records, trauma logs and triage notes as well as on-duty doctors and nurses. When a potential subject was identified and had been screened against primary inclusion/exclusion criteria, they were approached about study participation.<br><br>STUDY SITES<br>University of Cincinnati (UC)<br>Baylor College of Medicine (BCM)<br>University of Miami (UM)<br>University of California, San Francisco (UCSF)<br>University of Pennsylvania<br>University of Pittsburgh (UP)

	Massachusetts General Hospital (MGH)<br>The University of Texas Health Science Center at Houston
Outcomes	The primary neurological outcome measure is the Glasgow Outcome Scale– Extended (GOS-E) at 6 months following injury.
